# Hormone Replacement Therapy Does Not Eliminate Risk Factors for Joint Complications following Total Joint Arthroplasty: A Matched Cohort Study

**DOI:** 10.3390/pathophysiology30020011

**Published:** 2023-04-04

**Authors:** Lacee K. Collins, Matthew W. Cole, Timothy L. Waters, Michael Iloanya, Patrick A. Massey, William F. Sherman

**Affiliations:** 1 Department of Orthopaedic Surgery, Tulane University School of Medicine, New Orleans, LA 70112, USA; 2 Department of Orthopaedic Surgery, Louisiana State University Health Sciences Center, Shreveport, LA 71103, USA

**Keywords:** arthroplasty, hip, hormone replacement therapy, complications, testosterone, estrogen

## Abstract

Aging causes a reduction in testosterone and estrogen, which is linked to diminished bone mineral density. Hormone replacement therapy and its effect on the outcome of joint arthroplasties is unclear. The purpose of this study was to analyze the impact of testosterone replacement therapy (TRT) and estrogen replacement therapy (ERT) on the medical and joint outcomes of total hip (THA) and total knee arthroplasties (TKA). A retrospective cohort study was conducted using the PearlDiver database. Patients who received TRT or ERT perioperatively were matched to controls. Rates of 90-day medical complications and 2-year joint complications were queried. Patients who received TRT had an increased risk of revision, periprosthetic joint infection, and pooled joint complications within 2 years following a THA and increased rates of septic and aseptic revisions, and aseptic loosening after TKA compared to the control cohort. Patients receiving ERT had increased rates of aseptic loosening and pooled joint complications within 2 years following THA and increased rates of all-cause revisions and pooled joint complications after TKA. Patients who received TRT demonstrated significantly higher rates of revision rates and PJI. Patients who received perioperative ERT were significantly more likely to have increased risks of revision rates and joint infections.

## 1. Introduction

Aging is often accompanied by a loss of sex hormones, including testosterone and estrogen, leading to a variety of symptoms [1]. Serum testosterone levels decrease by 1% annually past the age of 40 in male patients [2]. Diminished muscle mass and strength, increased central body fat, erectile dysfunction, and fatigue are some of the most easily discernible clinical signs of relative androgen deficiency in men [2]. Likewise, estrogen levels peak in the mid-to-late 20s and decline by 50% by the age of 50 in females [3]. Low estrogen levels are accompanied by symptoms such as hot flashes, night sweats, fatigue, decreased libido, and atrophic vaginitis, among others [4]. Of particular concern to musculoskeletal health are decreases in testosterone and estrogen, which have both been linked to diminished bone mineral density, leading to an increased risk of fractures [5,6].

As such, hormone replacement therapy (HRT) has become a popular option for men and women to reduce these symptoms [7,8]. The reported benefits of testosterone replacement therapy (TRT) include improvements in bone density, muscle mass, body composition, sexual function and libido, mood, erythropoiesis, and quality of life [9]. Over half of testosterone prescriptions are written by primary care physicians (PMC3788396), the majority being given to older men with age-related recession in testosterone [10,11]. As a result, the use of testosterone is becoming increasingly prevalent, with approximately 2.3 million American men receiving TRT in 2013 [11]. Additionally, nearly 1.5 million women between the ages of 45 and 55 experience negative symptoms due to estrogen deficiencies [4]. Therefore, estrogen replacement therapy (ERT) has also become a common treatment option for menopausal women and has been found to significantly improve sleep, sexual dysfunction, mood symptoms, vasomotor symptoms, and quality of life [8]. Both TRT and ERT also have the benefits of normalizing bone turnover and preserving bone mineral density [12,13]. 

Although physiologic testosterone and estrogen have both been shown to have a positive effect on bone, the relationship between HRT and the outcomes of joint arthroplasties is unclear. Given the increasing utilization of both arthroplasty and HRT in older patients, the effect of HRT on joint arthroplasties is a crucial consideration for orthopaedic surgeons as they manage end-state arthritis. The purpose of this study was to analyze the impact of testosterone replacement therapy (TRT) in men and estrogen replacement therapy (ERT) in women on 90-day postoperative medical complications and 2-year joint outcomes of total hip (THA) and total knee arthroplasties (TKA).

## 2. Materials and Methods

### 2.1. Data Source and Study Design

Patient records were queried from the PearlDiver Mariner Database (PearlDiver Inc., Colorado Springs, CO, USA), a commercially available administrative claims database that contains deidentified patient data from the inpatient and outpatient settings. The database contains the medical records of patients across the United States from 2010 through Q1 of 2021, which were collected by an independent data aggregator. This study utilized the “M151Ortho” dataset within PearlDiver, which contains a random sample of 151 million patients. All health insurance payors are represented, including commercial, private, and government plans. Researchers extract the data using Current Procedural Technology (CPT) and the International Classification of Diseases, Ninth and Tenth Revision (ICD-9/ICD-10) codes. Institutional Review Board exemption was granted as the provided data was deidentified and compliant with the Health Insurance Portability and Accountability Act. No outside funding was received for this study.

A retrospective cohort study was conducted to investigate the impact of TRT and ERT on complication rates following primary total joint arthroplasty. THA and TKA were defined with CPT and associated ICD-9/10 procedural codes. To isolate primary THA, exclusion criteria were patients with a record of prior hemiarthroplasty, revision surgery, or diagnosis codes reflecting the presence of an artificial hip joint. All codes used to define inclusion and exclusion criteria are provided in Supplementary Materials. Additionally, patients with hip avascular necrosis, pathologic hip fractures, hip infectious processes, or conversion from prior hip surgery (i.e., CPT-27132) at the time of the primary THA were excluded. Finally, to ensure that postoperative complications were tied to the index THA, patients with contralateral hemiarthroplasty or THA during the two-year follow-up were also excluded. Likewise, to isolate primary TKA, exclusion criteria were patients with a prior diagnosis of an artificial knee joint, revision TKA, or other knee reconstructive procedures, as well as those with knee infection, fracture, or conversion procedures on the same day as the TKA. Additionally, those with contralateral primary TKA or unicompartmental TKA were also excluded. 

Subsequently, patients with TRT and ERT 6 months before and 6 months after index arthroplasty were identified by claims containing relevant drug codes. To limit potential transfer bias due to patients leaving or joining the dataset during the study period, only patients with continuous database enrollment for at least six months before and two years after the index arthroplasty were included. Additionally, in the ERT cohort, patients with concomitant progesterone use were excluded. 

### 2.2. Demographic Data and Clinical Characteristics

Baseline demographic data were obtained for all patient cohorts, including age, body mass index (BMI), year of arthroplasty, and U.S. region. BMI data were queried using ICD-9/10 diagnosis codes. As TRT is primarily administered to men, both the study and control cohorts were filtered to include only male patients. Likewise, as ERT is primarily administered to women, both the study and control cohorts for these comparisons were filtered to include only female patients. Clinical characteristics obtained included the length of stay (LOS) during the primary arthroplasty procedure, the prevalence of diabetes mellitus, tobacco use, osteoporosis, coronary artery disease, congestive heart failure, glucocorticoid use, rheumatoid arthritis, and obesity.

### 2.3. Outcomes

Rates of medical complications during the index hospital encounter and within 90 days were obtained postoperatively. Medical complications queried included inpatient readmissions, deep vein thrombosis (DVT), pulmonary embolism (PE), acute myocardial infarction (MI), acute kidney injury (AKI), blood transfusions, and inpatient readmissions. The codes used to define medical complications are outlined in the Supplementary Materials.

Joint complications were evaluated at two years postoperatively. Specific complications queried for THA included all-cause revision, prosthetic joint infections (PJI), prosthetic dislocation, aseptic loosening, and periprosthetic fracture. All-cause revision THA included revision of the femoral and/or acetabular components, liner exchange, implant removal, and insertion/removal of an antibiotic spacer. Hip PJI was defined as a two-stage revision for PJI, with the second stage defined as a conversion of prior hip surgery (i.e., CPT-27132 and associated ICD-9/10 codes) with concomitant removal of an antibiotic spacer. Codes used to define THA complications are provided in the Supplementary Materials.

Likewise, joint complications following the TKA were also evaluated. These complications included aseptic, septic, and all-cause revision, manipulation under anesthesia/lysis of adhesions for stiffness, aseptic prosthetic loosening, and periprosthetic fractures. These were defined using CPT and ICD9/10 codes. Codes used to define TKA complications are provided in the Supplementary Materials.

### 2.4. Statistical Analysis

Statistical analyses were performed using R statistical software (version 4.1.0; R Project for Statistical Computing, Vienna, Austria) integrated within the PearlDiver software with an α level set to 0.05. To reduce confounding bias, exact matching with patients who have never undergone a primary knee or hip replacement was performed to generate similar patient cohorts. Patients who received TRT and patients who received ERT were matched at a 1:4 ratio with controls on the following parameters: age, year of surgery, depression, rheumatoid arthritis, osteoporosis, chronic kidney disease, coronary artery disease, congestive heart failure, diabetes mellitus, tobacco use, and obesity. Additionally, in the ERT cohort, patients with a diagnosis of breast cancer during the study period were controlled for in the multivariate logistic regressions. 

Categorical variables were compared with a chi-square test, and continuous variables were compared with Welch’s *t* test or the Mann-Whitney U test. The rates of postoperative complications after primary TJA were compared using multivariable logistic regression adjusting for age, BMI, U.S. region, coronary artery disease, osteoporosis, rheumatoid arthritis, glucocorticoid use, tobacco use, and depression. Additionally, in the TRT cohort, regressions were controlled for the diagnosis of low testosterone based on CMS diagnosis codes. In the ERT cohort, regressions were controlled for breast cancer. Odds ratios (OR) with corresponding 95% confidence intervals (CIs) were calculated for each outcome. 

## 3. Results

### 3.1. THA-TRT Study Population

After exclusion criteria were applied, a total of 549,176 patients were identified who underwent primary THA. Of that group, 6863 patients received TRT. After 1:4 matching, 6725 patients who received TRT were matched with 26,698 controls. The two cohorts were statistically comparable in most matched parameters, indicating successful matching. Significant differences were found in regional distribution, rate of depression, rate of osteoporosis, coronary artery disease, CMS-diagnosed low testosterone, and in patients with a BMI of 35–40 (Table 1).

### 3.2. THA-ERT Study Population

After exclusion criteria were applied to the THA cohort, a total of 6302 patients were identified who also received ERT perioperatively. After 1:4 matching, 6302 patients who received ERT were matched to 25,127 controls. The two cohorts were statistically comparable in most matched parameters, indicating successful matching. Significant differences were found in regional distribution, rates of depression, rheumatoid arthritis, and osteoporosis (Table 2).

### 3.3. TKA-TRT Study Population

A total of 1,105,975 patients were identified who underwent primary TKA after exclusion criteria were applied to the TKA cohort found above, and a total of 14,445 patients were identified who also received TRT. After 1:4 matching, 14,290 patients who received TRT were matched to 57,002 controls. The two cohorts were statistically comparable in most matched parameters, indicating successful matching. Significant differences were found in regional distribution, coronary artery disease, depression, osteoporosis, and CMS low testosterone (Table 3).

### 3.4. TKA-ERT Study Population

After exclusion criteria were applied to the TKA cohort, a total of 16,525 patients were identified who also received ERT. After 1:4 matching, 16,525 patients who received ERT were matched to 65,952 controls. The two cohorts were statistically comparable in most matched parameters, indicating successful matching. Significant differences were found in the regional distribution of BMI < 30, BMI > 40, rheumatoid arthritis, depression, and osteoporosis (Table 4).

### 3.5. Complications after Primary THA in the TRT Cohort

Within 90 days following primary THA, patients who were receiving TRT exhibited significantly higher rates of acute kidney injury (3.54% vs. 2.61%; OR 1.22; 95% CI, 1.01–1.47). Inpatient readmissions were statistically lower in the TRT cohort (3.00% vs. 3.47%; OR 0.78; 95% CI, 0.64–0.93). All other 90-day medical complications were found to be not statistically different between the two groups (all *p* > 0.05). However, overall pooled 90-day medical complications were statistically higher in the TRT cohort (11.41% vs. 9.26%; OR 1.18; 95% CI, 1.06–1.32). Length of stay was also significantly longer in the TRT cohort (3.5 vs. 2.5 days, *p* < 0.001) (Table 5).

Within 2 years following primary THA, the rates of revision (3.57% vs. 2.34%; OR 1.46; 95% CI, 1.21–1.75) and PJI (2.16% vs. 1.43%; OR 1.67; 95% CI, 1.31–2.09) were statistically higher in the TRT cohort than controls. Additionally, overall pooled joint complications combined were significantly higher in the TRT group versus controls (5.65% vs. 3.45%; OR 1.72; 95% CI 1.48–2.00).

### 3.6. Complications after Primary THA in the ERT Cohort

Within 90 days following primary THA, patients who received ERT exhibited significantly lower rates of inpatient readmissions (3.65% vs. 4.06%; OR 0.85; 95% CI, 0.75–0.95), however, had significantly higher rates of pulmonary embolism following THA (0.78% vs. 0.57%; OR 1.40; 95% CI, 1.00–1.91). The length of stay was also significantly longer in the ERT cohort (7.9 vs. 3.1 days, *p* < 0.001) (Table 6).

Within 2 years following primary THA, patients who received ERT exhibited a significantly increased risk of aseptic loosening (0.81% vs. 0.55% OR 1.45; 95% CI, 1.04–2.00). Those in the ERT cohort also experienced significantly higher rates of combined joint complications compared to the controls (5.82% vs. 4.18%; OR 1.40; 95% CI, 1.24–1.58).

### 3.7. Complications after Primary TKA in the TRT Cohort

Within 90 days following primary TKA, patients who received TRT exhibited significantly higher rates of AKI (3.91% vs. 2.80%; OR 1.24; 95% CI, 1.10–1.40). However, the TRT cohort displayed significantly lower rates of inpatient readmission (4.85% vs. 5.55%; OR 0.77; 95% CI, 0.70–0.86) and transfusions (2.09% vs. 2.78%; OR 0.74; 95% CI, 0.64–0.86). The length of stay was also significantly longer in the TRT cohort (6.5 days vs. 2.7 days, *p* < 0.001) (Table 7).

Within 2 years following primary TKA, patients who received TRT exhibited significantly higher rates of septic revision (1.40% vs. 0.95%; OR 1.44; 95% CI, 1.19–1.76), aseptic revision (3.05% vs. 2.48%; OR 1.20; 95% CI, 1.05–1.37), all-cause revision (3.62% vs. 2.78%; OR 1.27; 95% CI, 1.13–1.44), periprosthetic fracture (0.27% vs. 0.21%; OR 1.53; 95% CI, 1.00–2.29), and aseptic loosening (0.97% vs. 0.73%; OR 1.34; 95% CI, 1.06–1.69). However, they also had significantly less instances of manipulation under anesthesia/lysis of adhesions (4.30% vs. 4.63%; OR 0.90; 95% CI, 0.81–1.00).

### 3.8. Complications after Primary TKA in the ERT Cohort

Within 90 days following primary TKA, patients who received ERT displayed significantly lower rates of DVT (0.24% vs. 0.32%; OR 0.63; 95% CI, 0.44–0.08), transfusions (4.82% vs. 5.57%; OR 0.85; 95% CI, 0.79–0.92), inpatient readmissions (4.94% vs. 5.69%; OR 0.83; 95% CI, 0.77–0.90), and pooled medical complications (11.07% vs. 11.79%; OR 0.91; 95% CI, 0.86–0.96). However, those in the ERT cohort displayed higher rates of MI compared to the control group (1.75% vs. 1.46%; OR 1.21; 95% CI, 1.05–1.38). The length of stay was also significantly longer in the ERT cohort (13.9 days vs. 2.9 days, *p* < 0.001) (Table 8).

Within 2 years following primary TKA, patients who received ERT exhibited significantly higher rates of septic revision (0.85% vs. 0.48 $; OR 1.70; 95% CI, 1.38–2.07), aseptic revision (2.04% vs. 1.74%; OR 1.18; 95% CI, 1.04–1.33) all-cause revision (2.51% vs. 1.92%; OR 1.31; 95% CI, 1.17–1.47), and pooled joint complications (7.20% vs. 6.44%; OR 1.12; 95% CI, 1.04–1.20).

## 4. Discussion

The results of this study demonstrate that taking TRT is associated with significantly higher rates of all-cause joint revisions and prosthetic joint infection revisions following THA and significantly higher rates of septic revision, all-cause revision, periprosthetic fractures, and aseptic loosening following TKA, as well as any joint complications overall in both groups than matched controls. Prior literature has demonstrated that the overall effect of testosterone in major joints is inconclusive. Tracz et al. studied bone density in different bones, finding that intramuscular testosterone caused moderately increased lumbar bone density, but the results were not significant for femoral neck bone density [14]. Additionally, Zhang et al. gathered data from 52 randomized control trials and found that, when compared to placebos, testosterone supplementation did not increase bone mineral density in men nor did it decrease the risk of fracture [15]. However, these results counter studies that have previously indicated that testosterone replacement therapy improves bone density in males with hypogonadal osteoporosis [16]. Tirabassi et al. demonstrated that bone mineral density slightly improves in men who receive testosterone replacement therapy [17]. Polackwich et al. demonstrated that bone mineral density did not significantly increase with the use of TRT after 6 months, but a longer length of treatment up to three years did find improvements [18].

The impact of TRT on bone health is controversial compared to other benefits of TRT, and this study demonstrated that TRT is associated with poorer outcomes following TJA. This does not imply that TRT causes poor TJA outcomes. The increased risk for joint complications in patients undergoing TRT, as demonstrated by the present study, may be attributable to chronic low testosterone in the years leading up to the patients TJA. It has been proven that bone growth and maintenance are significantly affected by testosterone levels, with testosterone levels below 300 ng/dL causing increased risk for bone loss and fracture [9,18]. Additionally, it has been demonstrated that a longer course of treatment with TRT may provide more benefit to bone health than shorter lengths of treatment [19]. The patients in our study’s TRT cohort were included if they were noted to have been given TRT within 6 months prior to the primary TJA or within 6 months post-operatively, which has been demonstrated to be the critical period affecting outcomes of TJA. Therefore, the patients included in the cohort who have been receiving TRT for a shorter period may not have experienced the benefits on bone health that other patients with a longer treatment course may have experienced. Additionally, Xue et al. demonstrated that peak femoral neck bone mineral density peaks at 20.5 years old and 20.1 years old in males and females, respectively [20]. As such, patients in the TRT cohort may have been experiencing diminishing BMD for an extended period prior to an official diagnosis of testicular hypofunction and the initiation of TRT. It is possible that our TRT cohort had a long, undiagnosed history of low BMD without a sufficiently long duration of TRT to remediate this deficit.

Likewise, in patients who were receiving ERT, the chronic decline of estrogen levels can be linked to their overall poor bone health leading up to the primary TJA. Secretion of dehydroepiandrosterone (DHEA), a precursor to estrogen, reduces with aging, with levels decreasing to only 10% in the elderly, relative to the peak concentration [21]. Khosla et al. demonstrated that estrogen is a major regulator of bone metabolism and has a significantly protective effect on bone health [22]. With declining estrogen levels in post-menopausal women, bone resorption outpaces bone formation and leads to an increased risk for the development of osteoporosis [22,23]. ERT has been found to preserve BMD at skeletal sites, including the femoral neck [12]. Similar to the effects of testosterone, Bagger et al. found that ERT in women could take up to three years to provide benefit to bone mass and reduce the risks of osteoporotic fractures [24]. As this study focused only on the effects of perioperative use of estrogen, patients in the ERT cohort may not have been taking ERT long enough to fully impact bone health. 

The present study also demonstrated that prosthetic joint infections following THA and septic revisions following TKA occurred at significantly higher rates in the TRT cohort than in controls. This increased infection rate may be related to testosterone’s negative effect on the immune system. Furman et al. demonstrated that testosterone played an immunosuppressive role in patients receiving the influenza vaccination [25]. Salciccia et al. also supported this by demonstrating that testosterone acts negatively on the immune response in both bacterial and viral infections [26]. Additionally, studies have found an increased risk of infection from TRT at the site of injection. Hope et al. found that 36% of the 1058 patients in the study reported injection site infections during the year-long study [27]. The current study’s finding that infection rates were higher in the TRT cohort aligns with the literature that implicates the testosterone relationship with a diminished immune response and TRT with increased rates of injection site infections. However, topical testosterone was included in the TRT cohort, thereby eliminating injection site infections from that fraction of patients.

While TRT may play a long-term role in bone health, there may also be effects on other organs, complicating the perioperative period. From a medical management standpoint, this study’s finding of higher rates of acute kidney injury in the TRT cohort is of great value when surveilling patients in the TJA postoperative period. The literature is conflicting regarding testosterone’s effect on dilation or constriction of arterial vessels. Herring et al. explain that testosterone may have an ability to attenuate vasodilation and intensify constriction of blood vessels, especially in the presence of other vasodilatory compounds [28]. Vasoconstriction of the renal afferent arterioles in the setting of TRT could explain the increased rates of acute kidney injury observed in our TRT cohort. 

The present study demonstrated that among those receiving ERT, there was an overall significant decrease in the incidence of medical complications following TKA. It was also demonstrated that there were decreases in rates of transfusion and inpatient readmissions. The literature is not well researched regarding the perioperative use of ERT in patients. Nussmeier et al. studied the perioperative use of HRT in women undergoing coronary artery bypass grafting and found no increased risk of any adverse outcomes [29]. Additionally, it was demonstrated in this study that the rate of pulmonary embolism was significantly higher in the ERT cohort who underwent THA. This is consistent with the literature regarding ERT use, which has demonstrated a significantly increased risk of thromboembolic disease in both oral and topical estrogen therapy [8]. However, in those who underwent TKA and were receiving perioperative ERT, the rate of deep vein thromboses was significantly lower, which is contrary to what was seen in the THA cohort. However, as some data regarding these medical outcomes is both surprising and conflicting, it is evident that more research should be completed to better evaluate the impact of HRT on postoperative outcomes. 

### Limitations

There are several limitations to this study. First, by only evaluating complications within two years, this analysis is limited to short-term outcomes. Furthermore, because continuous database enrollment for two years after arthroplasty was required for inclusion, patients who died within two years after surgery were excluded. Therefore, these results may not apply to patients with a high perioperative mortality risk. Additionally, the possibility of coding errors is inherent in any analysis of administrative claims data. However, such instances are rare and made up only 0.7% of Medicare and Medicaid payments in 2021 [30]. Since this analysis relied on claims data, it is possible that uncharted complications were not captured. The database also does not contain data on patients’ BMD (e.g., T-score), which prevented the characterization of bone health in the included population. Though a large patient database was analyzed, this result may reflect inadequate power; future analyses of larger samples of patients who have received HRT and are undergoing TJA are warranted. Additionally, although exact matching and multivariable regression were used, other confounders could have influenced the results. BMI data was also not universally available for all included patients, and therefore the adjustment for BMI was incomplete. Lastly, this study also did not include information regarding the length of testosterone or estrogen replacement therapy for the study cohorts, as the purpose was to look solely at the perioperative use of HRT. This limits this study’s ability to determine the influence of chronic HRT versus a more recent initiation of HRT. 

## 5. Conclusions

Overall, patients who required HRT were more likely to have medical complications after TJA. Patients who received perioperative TRT had significantly higher joint complications following TJA, including higher rates of revision, PJI, and periprosthetic fractures. Additionally, patients with perioperative use of ERT were also likely to experience significantly higher rates of joint complications, including infections and revision rates. The increase in joint complications may be due to the concept that older patients who require HRT may have had diminishing bone health secondary to low estrogen or testosterone in the years leading up to initiation of HRT. This demonstrates that this diminished bone health may not be adequately compensated by HRT, resulting in greater joint complications post-operatively.

## Figures and Tables

**Table 1:** Demographics for THA-TRT vs. controls.

	TRT (*n* = 6725)		Controls (*n* = 26,698)		*p*-Value
Characteristics	*n*	%	*n*	%	
Age (Years), Mean ± SD	61.7 ± 8.6	-	61.9 ± 8.5	-	0.48
U.S. Region, *n* (%)					
Northeast	1163	17.3%	6417	24.0%	<0.001
South	2913	43.3%	8609	32.2%	<0.001
Midwest	1451	21.6%	7689	28.8%	<0.001
West	1204	17.9%	3892	14.6%	<0.001
BMI, *n* (%)					
<30	132	2.0%	498	1.9%	0.46
30–35	191	2.8%	726	2.7%	0.38
35–40	112	1.7%	557	2.1%	0.03
>40	139	2.1%	540	2.0%	0.67
Comorbidities, *n* (%)					
Diabetes Mellitus	3189	47.4%	12,645	47.4%	0.94
Obesity	3460	51.4%	13,719	51.4%	0.94
Rheumatoid Arthritis	281	4.2%	1008	3.8%	0.13
Coronary Artery Disease	2604	38.7%	9960	37.3%	0.03
Congestive Heart Failure	647	9.6%	2532	9.5%	0.75
Glucocorticoid Use	1123	16.7%	4334	16.2%	0.37
Depression	2554	38.0%	7608	28.5%	<0.001
Osteoporosis	293	4.4%	857	3.2%	<0.001
Low T	3441	51.2%	1048	3.9%	<0.001
Tobacco Use	2829	42.1%	11,218	42.0%	0.95
Length of Stay (Days), Mean ± SD	3.5 ± 2.4	-	2.5 ± 1.6	-	<0.001

Bolded OR (95% CI)/*p* values indicate statistically significant results.

**Table 2:** Demographics for THA-ERT vs. controls.

	ERT (*n* = 6302)		Controls (*n* = 25,127)		*p*-Value
Characteristics	*n*	%	*n*	%	
Age (Years), Mean ± SD	66.6 ± 7.8	-	66.6 ± 7.7	-	0.82
U.S. Region, *n* (%)					
Northeast	1029	16.3%	5446	21.7%	<0.001
South	2455	39.0%	8627	34.3%	<0.001
Midwest	1632	25.9%	6975	27.8%	0.003
West	1172	18.6%	3992	15.9%	<0.001
BMI, *n* (%)					
<30	186	3.0%	726	2.9%	0.06
30–35	117	1.9%	444	1.8%	0.10
35–40	57	0.9%	318	1.3%	0.09
>40	65	1.0%	387	1.5%	0.01
Comorbidities, *n* (%)					
Diabetes Mellitus	2073	32.9%	8255	32.9%	0.96
Obesity	2130	33.8%	8488	33.8%	0.99
Rheumatoid Arthritis	550	8.7%	1987	7.9%	0.03
Coronary Artery Disease	1778	28.2%	7101	28.3%	0.95
Congestive Heart Failure	550	8.7%	2344	9.3%	0.15
Glucocorticoid Use	1070	17.0%	4216	16.8%	0.72
Depression	2972	47.2%	10,459	41.6%	<0.001
Osteoporosis	1135	18.0%	4802	19.1%	0.05
Breast Cancer	113	1.8%	422	1.7%	0.57
Tobacco Use	2141	34.0%	8518	33.9%	0.92
Length of Stay (Days), Mean ± SD	7.9 ± 13.1	-	3.1 ± 5.7	-	<0.001

Bolded OR (95% CI)/*p* values indicate statistically significant results.

**Table 3:** Demographics for TKA-TRT vs. controls.

	TRT (*n* = 14,290)		Controls (*n* = 57,002)		*p*-Value
Characteristics	*n*	%	*n*	%	
Age (Years), Mean ± SD	62.6 ± 8.0	-	62.6 ± 8.0	-	0.57
U.S. Region, *n* (%)					
Northeast	2011	14.1%	11,117	19.5%	<0.001
South	6695	46.9%	20,683	36.3%	<0.001
Midwest	3187	22.3%	16,777	29.4%	<0.001
West	2388	16.7%	8245	14.5%	<0.001
BMI, *n* (%)					
<30	249	1.7%	983	1.7%	0.99
30–35	375	2.6%	1589	2.8%	0.17
35–40	345	2.4%	1304	2.3%	0.39
>40	389	2.7%	1503	2.6%	0.53
Comorbidities, *n* (%)					
Diabetes Mellitus	7840	54.9%	31,271	54.9%	1.00
Obesity	8000	56.0%	31,916	56.0%	0.99
Rheumatoid Arthritis	712	5.0%	2632	4.6%	0.07
Coronary Artery Disease	6219	43.5%	23,665	41.5%	<0.001
Congestive Heart Failure	1565	11.0%	6188	10.9%	0.75
Glucocorticoid Use	2475	17.3%	9778	17.2%	0.65
Depression	5703	39.9%	17,420	30.6%	<0.001
Osteoporosis	525	3.7%	1728	3.0%	<0.001
CMS Low T	7265	50.8%	2507	4.4%	<0.001
Tobacco Use	6372	44.6%	25,406	44.6%	0.97
Length of Stay (Days), Mean ± SD	6.5 ± 3.67	-	2.7 ± 1.55	-	<0.001

Bolded OR (95% CI)/*p* values indicate statistically significant results.

**Table 4:** Demographics for TKA-ERT vs. controls.

	ERT (*n* = 16,525)		Controls (*n* = 65,952)		*p*-Value
Characteristics	*n*	%	*n*	%	
Age (Years), Mean ± SD	65.5 ± 8.0	-	62.6 ± 8.0	-	0.73
U.S. Region, *n* (%)					
Northeast	2210	13.4%	12,483	18.9%	<0.001
South	7225	43.7%	25,069	38.0%	<0.001
Midwest	4384	26.5%	18,706	28.4%	<0.001
West	2670	16.2%	9459	14.3%	<0.001
BMI, *n* (%)					
<30	445	2.7%	1397	2.1%	<0.001
30–35	381	2.3%	1407	2.1%	0.01
35–40	254	1.5%	1238	1.9%	0.02
>40	315	1.9%	1867	2.8%	<0.001
Comorbidities, *n* (%)					
Diabetes Mellitus	6819	41.3%	27,206	41.3%	0.98
Obesity	7571	45.8%	30,211	45.8%	1.00
Rheumatoid Arthritis	1652	10.0%	6011	9.1%	<0.001
Coronary Artery Disease	4896	29.6%	19,828	30.1%	0.28
Congestive Heart Failure	1520	9.2%	6716	10.2%	<0.001
Glucocorticoid Use	3099	18.8%	12,274	18.6%	0.68
Depression	8424	51.0%	29,899	45.3%	<0.001
Osteoporosis	2743	16.6%	11,788	17.9%	<0.001
Breast Cancer	1144	6.9%	4480	6.8%	0.56
Tobacco Use	5113	30.9%	20,364	30.9%	0.88
Length of Stay (Days), Mean ± SD	13.9 ± 6.5	-	2.9 ± 1.4	-	<0.001

Bolded OR (95% CI)/*p* values indicate statistically significant results.

**Table 5:** Overall complications in the THA-TRT cohort vs. controls.

	TRT		Controls		Statistical Analysis
	(*n* = 6725)		(*n* = 26,698)		(Ref Group, TRT cohort)
Complication	*n*	%	*n*	%	OR (95% CI)
90 Days					
Any Medical Complication	767	11.4%	2472	9.3%	1.18 (1.06–1.32)
DVT	38	0.6%	94	0.4%	1.24 (0.77–1.98)
PE	48	0.7%	168	0.6%	1.02 (0.68–1.50)
AKI	238	3.5%	696	2.6%	1.22 (1.01–1.47)
MI	228	3.4%	889	3.3%	0.96 (0.80–1.15)
Transfusion	205	3.0%	918	3.4%	0.90 (0.74–1.07)
Inpatient Readmission	202	3.0%	927	3.5%	0.78 (0.64–0.93)
2 Years					
Any Joint Complication	380	5.7%	920	3.4%	1.72 (1.48–2.00)
Dislocation	83	1.2%	243	0.9%	1.24 (0.91–1.68)
Revision THA	240	3.6%	624	2.3%	1.46 (1.21–1.75)
PJI	145	2.2%	381	1.4%	1.67 (1.31–2.09)
Aseptic Loosening	50	0.7%	168	0.6%	0.84 (0.56–1.25)
Periprosthetic Fracture	39	0.6%	129	0.5%	1.01 (0.64–1.56)

Bolded OR (95% CI)/*p* values indicate statistically significant results.

**Table 6:** Overall complications in the THA-ERT cohort vs. controls.

	ERT		Controls		Statistical Analysis
	(*n* = 6302)		(*n* = 25,127)		(Ref Group, ERT cohort)
Complication	*n*	%	*n*	%	OR (95% CI)
90 Days					
Any Medical Complication	824	13.1%	3314	13.2%	0.96 (0.88–1.05)
DVT	12	0.2%	59	0.2%	0.70 (0.36–1.27)
PE	49	0.8%	142	0.6%	1.40 (1.00–1.91)
AKI	116	1.8%	455	1.8%	0.96 (0.77–1.18)
MI	124	2.0%	425	1.7%	1.15 (0.93–1.41)
Transfusion	494	7.8%	2117	8.4%	0.91 (0.92–1.01)
Inpatient Readmission	230	3.6%	1019	4.1%	0.85 (0.75–0.95)
2 Years					
Any Joint Complication	367	5.8%	1050	4.2%	1.40 (1.24–1.58)
Dislocation	122	1.9%	402	1.6%	1.18 (0.96–1.45)
Revision THA	202	3.2%	720	2.9%	1.11 (0.94–1.30)
PJI	78	1.2%	302	1.2%	1.01 (0.78–1.29)
Aseptic Loosening	51	0.8%	139	0.6%	1.45 (1.04–2.00)
Periprosthetic Fracture	59	0.9%	241	1.0%	0.97 (0.72–1.29)

Bolded OR (95% CI)/*p* values indicate statistically significant results.

**Table 7:** Overall complications in the TKA-TRT cohort vs. controls.

	TRT		Controls		Statistical Analysis
	(*n* = 14,290)		(*n* = 57,002)		(Ref Group, TRT cohort)
Complication	*n*	%	*n*	%	OR (95% CI)
90 Days					
Any Medical Complication	1798	12.6%	6950	12.2%	0.93 (0.87–1.00)
DVT	58	0.4%	212	0.4%	0.74 (0.51–1.06)
PE	143	1.0%	508	0.9%	0.95 (0.75–1.19)
AKI	559	3.9%	1595	2.8%	1.24 (1.10–1.40)
MI	525	3.7%	1910	3.4%	1.03 (0.91–1.16)
Transfusion	299	2.1%	1584	2.8%	0.74 (0.64–0.86)
Inpatient Readmission	693	4.8%	3164	5.6%	0.77 (0.70–0.86)
2 Years					
Any Joint Complication	1151	8.1%	4257	7.5%	1.05 (0.97–1.14)
Septic Revision	200	1.4%	542	1.0%	1.44 (1.19–1.76)
Aseptic Revision	436	3.1%	1414	2.5%	1.20 (1.05–1.37)
All-Cause Revision	518	3.6%	1585	2.8%	1.27 (1.13–1.44)
Periprosthetic fracture	39	0.3%	117	0.2%	1.53 (1.00–2.29)
Stiffening	615	4.3%	2641	4.6%	0.90 (0.81–1.00)
Loosening	139	1.0%	416	0.7%	1.34 (1.06–1.69)

Bolded OR (95% CI)/*p* values indicate statistically significant results.

**Table 8:** Overall complications in the TKA-ERT cohort vs. controls.

	ERT		Controls		Statistical Analysis
	(*n* = 16,525)		(*n* = 65,952)		(Ref Group, ERT cohort)
Complication	*n*	%	*n*	%	OR (95% CI)
90 Days					
Any Medical Complication	1830	11.1%	7777	11.8%	0.91 (0.86–0.96)
DVT	39	0.2%	209	0.3%	0.63 (0.44–0.88)
PE	136	0.8%	615	0.9%	0.87 (0.72–1.05)
AKI	355	2.1%	1237	1.9%	1.20 (0.99–1.27)
MI	289	1.7%	960	1.5%	1.21 (1.05–1.38)
Transfusion	796	4.8%	3672	5.6%	0.85 (0.79–0.92)
Inpatient Readmission	816	4.9%	3750	5.7%	0.83 (0.77–0.90)
2 Years					
Any Joint Complication	1189	7.2%	4249	6.4%	1.12 (1.04–1.20)
Septic Revision	141	0.9%	319	0.5%	1.70 (1.38–2.07)
Aseptic Revision	337	2.0%	1149	1.7%	1.18 (1.04–1.33)
All-Cause Revision	415	2.5%	1266	1.9%	1.31 (1.17–1.47)
Periprosthetic fracture	47	0.3%	196	0.3%	0.92 (0.67–1.27)
Stiffening	747	4.5%	2879	4.4%	1.03 (0.95–1.12)
Loosening	118	0.7%	405	0.6%	1.12 (0.90–1.37)

Bolded OR (95% CI)/*p* values indicate statistically significant results.

## Data Availability

PearlDiver Mariner Database (PearlDiver Inc., Colorado Springs, CO, USA).

## Supplementary Materials

The following supporting information can be downloaded at: https://www.mdpi.com/article/10.3390/pathophysiology30020011/s1, Table S1: Codes used to define inclusion/exclusion criteria and other demographic and clinical variables; Table S2: Codes used to define medical complication outcomes; Table S3: Codes used to define THA joint complication outcomes; Table S4: Codes used to define TKA joint complication outcomes.
