# Supplementary material for: Hormone Replacement Therapy Does Not Eliminate Risk Factors for Joint Complications following Total Joint Arthroplasty: A Matched Cohort Study
Source: Pathophysiology. 2023 Apr 4;30(2):123–35. doi: 10.3390/pathophysiology30020011 (PMC10123744; doi:10.3390/pathophysiology30020011)
Supplement: Supplementary file 1 [file pathophysiology-30-00011-s001.zip › pathophysiology-2194992-supplementary.pdf]

Supplementary Materials: PearlDiver Codes

Table S1: Codes used to define inclusion/exclusion criteria and other demographic and clinical variables

Criteria	Code(s)
Inclusion Criteria	
THA	CPT-27130, ICD-9-P-8151, ICD-10-P-0SR9019, ICD-10-P-0SR901A, ICD-10-P-0SR901Z, ICD-10-P-0SR9029, ICD-10-P-0SR902A, ICD-10-P-0SR902Z, ICD-10-P-0SR9039, ICD-10-P-0SR903A, ICD-10-P-0SR903Z, ICD-10-P-0SR9049, ICD-10-P-0SR904A, ICD-10-P-0SR904Z, ICD-10-P-0SR9069, ICD-10-P-0SR906A, ICD-10-P-0SR906Z, ICD-10-P-0SR90J9, ICD-10-P-0SR90JA, ICD-10-P-0SR90JZ, ICD-10-P-0SRB019, ICD-10-P-0SRB01A, ICD-10-P-0SRB01Z, ICD-10-P-0SRB029, ICD-10-P-0SRB02A, ICD-10-P-0SRB02Z, ICD-10-P-0SRB039, ICD-10-P-0SRB03A, ICD-10-P-0SRB03Z, ICD-10-P-0SRB049, ICD-10-P-0SRB04A, ICD-10-P-0SRB04Z, ICD-10-P-0SRB069, ICD-10-P-0SRB06A, ICD-10-P-0SRB06Z, ICD-10-P-0SRB0J9, ICD-10-P-0SRB0JA, ICD-10-P-0SRB0JZ
TKA	CPT-27447, ICD-9-P-8154, ICD-10-P-0SRC069, ICD-10-P-0SRC06A, ICD-10-P-0SRC06Z, ICD-10-P-0SRC0J9, ICD-10-P-0SRC0JA, ICD-10-P-0SRC0JZ, ICD-10-P-0SRD069, ICD-10-P-0SRD06A, ICD-10-P-0SRD06Z, ICD-10-P-0SRD0J9, ICD-10-P-0SRD0JA, ICD-10-P-0SRD0JZ
Testosterone Replacement Therapy	DRUG-DEPO-TESTOSTERONE, DRUG-FIRST-TESTOSTERONE, DRUG-FIRST-TESTOSTERONE_MC, DRUG-METHYLTESTOSTERONE, DRUG-METHYLTESTOSTERONE_MICRONIZED, DRUG-TESTOSTERONE, DRUG-TESTOSTERONE_CYPIONATE, DRUG-TESTOSTERONE_CYPIONATE_MICRO, DRUG-TESTOSTERONE_ENANTHATE, DRUG-TESTOSTERONE_MICRONIZED, DRUG-TESTOSTERONE_PROPIONATE, GENERIC_DRUG-METHYLTESTOSTERONE, GENERIC_DRUG-METHYLTESTOSTERONE__MICRONIZED, GENERIC_DRUG-TESTOSTERONE, GENERIC_DRUG-TESTOSTERONE_CYPIONATE, GENERIC_DRUG-TESTOSTERONE_CYPIONATE__MICRO, GENERIC_DRUG-TESTOSTERONE_ENANTHATE, GENERIC_DRUG-TESTOSTERONE_MICRONIZED, GENERIC_DRUG-TESTOSTERONE_PROPIONATE, GENERIC_DRUG-TESTOSTERONE_UNDECANOATE, DRUG-ANDROGEL, DRUG-ANDRODERM, DRUG-FORTESTA, DRUG-AXIRON, DRUG-VOGELXO, DRUG-TESTIM
Estrogen Replacement Therapy	LC_ERT = OR {DRUG-DELESTROGEN, DRUG-CONJUGATED_ESTROGENS, GENERIC_DRUG-ESTROGENS__CONJUGATED, GENERIC_DRUG-ESTROGENS_CONJ/BAZEDOXIFENE, GENERIC_DRUG-ESTROGENS_CONJ._SYNTHETIC_A, GENERIC_DRUG-ESTROGENS_CONJ._SYNTHETIC_B, GENERIC_DRUG-

	ESTROGENS_CONJUGATED, GENERIC_DRUG-ESTROGENS_ESTERIFIED}
Exclusion Criteria	
Prior Hip Hemiarthroplasty	CPT-27125
Presence of Artificial Hip Joint	ICD-9-D-V4364, ICD-10-D-Z96641, ICD-10-D-Z96642, ICD-10-D-Z96643, ICD-10-D-Z96649
Avascular Necrosis Hip	ICD-9-D-73342, ICD-10-D-M87051, ICD-10-D-M87052, ICD-10-D-M87059
Conversion from Prior Hip Surgery	CPT-27132
Pathologic Fracture Hip	ICD-9-D-73314, ICD-9-D-73315, ICD-10-D-M84459A, ICD-10-D-M84559A, ICD-10-D-M84659A
Septic Arthritis Hip	ICD-9-D-71105, ICD-9-D-71106, ICD-9-D-71145, ICD-9-D-71146, ICD-10-D-M00851, ICD-10-D-M00852, ICD-10-D-M00859
Other	
Tobacco Use	ICD-9-D-3051, ICD-9-D-V1582, ICD-10-D-F17220, ICD-10-D-F17221, ICD-10-D-F17223, ICD-10-D-F17228, ICD-10-D-F17229, ICD-10-D-F17290, ICD-10-D-F17291, ICD-10-D-F17293, ICD-10-D-F17298, ICD-10-D-F17299, ICD-10-D-Z720
Diabetes Mellitus	ICD-9-D-24900:ICD-9-D-25099, ICD-9-D-7902, ICD-9-D-79021, ICD-9-D-79022, ICD-9-D-79029, ICD-9-D-7915, ICD-9-D-7916, ICD-10-D-E090:ICD-10-D-E139
Obesity	ICD-9-D-2780, ICD-9-D-27800, ICD-9-D-27801, ICD-9-D-27802, ICD-9-D-27803, ICD-10-D-E660:ICD-10-D-E669
Osteoporosis	ICD-10-D-M80822P!ICD-10-D-M80822S!ICD-10-D-M80029D!ICD-10-D-M80822K!ICD-10-D-M80029A!ICD-10-D-M80049A!ICD-10-D-M80869D!ICD-10-D-M80869A!ICD-10-D-M8008XG!ICD-10-D-M80821P!ICD-10-D-M80821S!ICD-10-D-M8008XK!ICD-10-D-M8008XA!ICD-10-D-M80821G!ICD-10-D-M80821K!ICD-10-D-M8008XD!ICD-10-D-M80822A!ICD-10-D-M80822D!ICD-10-D-M80069A!ICD-10-D-M8008XP!ICD-10-D-M8008XS!ICD-10-D-M80879A!ICD-10-D-M80879D!ICD-10-D-M80851A!ICD-10-D-M80039A!ICD-10-D-M80039D!ICD-10-D-M80812S!ICD-10-D-M80812D!ICD-10-D-M80839S!ICD-10-D-M80812A!ICD-10-D-M80811G!ICD-10-D-M80811S!ICD-10-D-M80061D!ICD-10-D-M80051A!ICD-10-D-M80061G!ICD-10-D-M80862D!ICD-10-D-M80852P!ICD-10-D-M80012P!ICD-10-D-M80852S!ICD-10-D-M80012S!ICD-10-D-M80862P!ICD-10-D-M80022P!ICD-10-D-M80852D!ICD-10-D-M80051G!ICD-10-D-M80811A!ICD-10-D-M80852G!ICD-10-D-M80051D!ICD-10-D-M80862K!ICD-10-D-M80022S!ICD-10-D-M80811D!ICD-10-D-M80061A!ICD-10-D-M80852K!ICD-10-D-M80079S!ICD-10-D-M80012D!ICD-10-D-M80022G!ICD-10-D-M80829D!ICD-10-D-M80051P!ICD-10-D-M80061S!ICD-10-D-M80051K!ICD-10-D-M80862S!ICD-10-D-M80022K!ICD-10-D-M810!ICD-10-D-M80012A!ICD-10-D-M80829A!ICD-10-D-M80079K!ICD-10-D-M80061K!ICD-10-D-M80022A!ICD-10-D-M80079G!ICD-10-D-M80051S!ICD-10-D-M80079A!ICD-10-D-M80022D!ICD-10-D-M80839A!ICD-10-D-M80061P!ICD-10-D-M80829G!ICD-10-D-M80079D!ICD-10-D-M80012K!ICD-10-D-M80039S!ICD-10-D-M80861G!ICD-10-D-M80052A!ICD-10-D-M80062D!ICD-10-D-M80851K!ICD-

	10-D-M80011S!ICD-10-D-M80049D!ICD-10-D-M80049K!ICD-10-D-M80861D!ICD-10-D-M80851P!ICD-10-D-M80062G!ICD-10-D-M80052K!ICD-10-D-M80039K!ICD-10-D-M80021P!ICD-10-D-M80861P!ICD-10-D-M80851D!ICD-10-D-M80052G!ICD-10-D-M80062A!ICD-10-D-M80039G!ICD-10-D-M80861K!ICD-10-D-M818!ICD-10-D-M80021S!ICD-10-D-M80851G!ICD-10-D-M80052D!ICD-10-D-M80879K!ICD-10-D-M80052S!ICD-10-D-M80062S!ICD-10-D-M80052P!ICD-10-D-M80011D!ICD-10-D-M80021G!ICD-10-D-M80011A!ICD-10-D-M80879P!ICD-10-D-M80861S!ICD-10-D-M80852A!ICD-10-D-M80021K!ICD-10-D-M80879S!ICD-10-D-M80862A!ICD-10-D-M80021A!ICD-10-D-M80011K!ICD-10-D-M80062K!ICD-10-D-M80851S!ICD-10-D-M80021D!ICD-10-D-M80011G!ICD-10-D-M80869S!ICD-10-D-M80072K!ICD-10-D-M80861A!ICD-10-D-M80072P!ICD-10-D-M80072S!ICD-10-D-M80072A!ICD-10-D-M80849S!ICD-10-D-M80072D!ICD-10-D-M80072G!ICD-10-D-M80071K!ICD-10-D-M80071P!ICD-10-D-M80071S!ICD-10-D-M80841A!ICD-10-D-M80821A!ICD-10-D-M80821D!ICD-10-D-M80829P!ICD-10-D-M80071D!ICD-10-D-M80071A!ICD-10-D-M80071G!ICD-10-D-M80829S!ICD-10-D-M8088XP!ICD-10-D-M8088XS!ICD-10-D-M8088XG!ICD-10-D-M8088XK!ICD-9-D-73302!ICD-9-D-73301!ICD-9-D-73303!ICD-10-D-M80859S!ICD-9-D-73300!ICD-10-D-M8080XS!ICD-10-D-M8080XP!ICD-10-D-M80831A!ICD-9-D-73309!ICD-10-D-M80831D!ICD-10-D-M80059A!ICD-10-D-M8000XD!ICD-10-D-M80019D!ICD-10-D-M8000XA!ICD-10-D-M80019A!ICD-10-D-M80872D!ICD-10-D-M8000XG!ICD-10-D-M80019G!ICD-10-D-M80842D!ICD-10-D-M80042P!ICD-10-D-M80029S!ICD-10-D-M80842A!ICD-10-D-M8000XK!ICD-10-D-M80042S!ICD-10-D-M80029P!ICD-10-D-M80059S!ICD-10-D-M80872G!ICD-10-D-M8000XP!ICD-10-D-M80832A!ICD-10-D-M80029K!ICD-10-D-M80032S!ICD-10-D-M80872K!ICD-10-D-M80872P!ICD-10-D-M80019S!ICD-10-D-M80832D!ICD-10-D-M8000XS!ICD-10-D-M80032P!ICD-10-D-M80841S!ICD-10-D-M80872S!ICD-10-D-M80069K!ICD-10-D-M80819A!ICD-10-D-M8080XA!ICD-10-D-M80059G!ICD-10-D-M80841P!ICD-10-D-M80819D!ICD-10-D-M80042D!ICD-10-D-M8080XD!ICD-10-D-M80831K!ICD-10-D-M80059D!ICD-10-D-M80032G!ICD-10-D-M80042A!ICD-10-D-M80042G!ICD-10-D-M8080XG!ICD-10-D-M80032D!ICD-10-D-M80069D!ICD-10-D-M80059P!ICD-10-D-M80032A!ICD-10-D-M80841G!ICD-10-D-M8080XK!ICD-10-D-M80042K!ICD-10-D-M80831S!ICD-10-D-M80841D!ICD-10-D-M80059K!ICD-10-D-M80871A!ICD-10-D-M80871D!ICD-10-D-M80859A!ICD-10-D-M80041P!ICD-10-D-M80871G!ICD-10-D-M80871K!ICD-10-D-M80031S!ICD-10-D-M80849A!ICD-10-D-M80859D!ICD-10-D-M80859G!ICD-10-D-M80871P!ICD-10-D-M80031P!ICD-10-D-M80832G!ICD-10-D-M8088XD!ICD-10-D-M80031K!ICD-10-D-M80871S!ICD-10-D-M80842S!ICD-10-D-M80832K!ICD-10-D-M80041A!ICD-10-D-M8088XA!ICD-10-D-M80031A!ICD-10-D-M80041D!ICD-10-D-M80031D!ICD-10-D-M80842K!ICD-10-D-M80832S!ICD-10-D-M80041K!ICD-10-D-M80872A!;

Coronary Artery Disease	ICD-9-D-4110:ICD-9-D-4149, ICD-10-D-I25:ICD-10-D-I259
Congestive Heart Failure	ICD-9-D-39891, ICD-9-D-4280, ICD-9-D-4281, ICD-9-D-42820, ICD-9-D-42821, ICD-9-D-42822, ICD-9-D-42823, ICD-9-D-42830, ICD-9-D-42831, ICD-9-D-42832, ICD-9-D-42833, ICD-9-D-42840, ICD-9-D-42841, ICD-9-D-42842, ICD-9-D-42843, ICD-9-D-4289, ICD-10-D-I150:ICD-10-D-I159
Glucocorticoid use	USC-52250,USC-52220,USC-52210, USC-62320
Rheumatoid Arthritis	ICD-9-D-7140, ICD-9-D-7142, ICD-10-D-M0520:ICD-10-D-M061
Progesterone use	DRUG-FIRST-PROGESTERONE_MC_5, DRUG-FIRST-PROGESTERONE_VGS_100, DRUG-FIRST-PROGESTERONE_VGS_200, DRUG-FIRST-PROGESTERONE_VGS_25, DRUG-FIRST-PROGESTERONE_VGS_400, DRUG-FIRST-PROGESTERONE_VGS_50, DRUG-HYDROXYPROGESTERONE_CAPROATE, DRUG-MEDROXYPROGESTERONE_ACETATE, DRUG-MEDROXYPROGESTERONE_AC_MICRO, DRUG-PROGESTERONE, DRUG-PROGESTERONE_IN_OIL, DRUG-PROGESTERONE_MICRONIZED, DRUG-PROGESTERONE_SUPP.KIT, GENERIC_DRUG-HYDROXYPROGESTERONE_CAPROATE, GENERIC_DRUG-MEDROXYPROGESTERONE_ACETATE, GENERIC_DRUG-MEDROXYPROGESTERONE_AC__MICRO, GENERIC_DRUG-PROGESTERONE, GENERIC_DRUG-PROGESTERONE_MICRONIZED, GENERIC_DRUG-PROGESTERONE__MICRONIZED
Breast cancer	

Table S2. Codes used to define medical complication outcomes

Criteria	Code(s)
Medical Complications	
Deep Vein Thrombosis	ICD-9-D-4532, ICD-9-D-4533, ICD-9-D-4534, ICD-9-D-45382, ICD-9-D-45384, ICD-9-D-45385, ICD-9-D-45386, ICD-10-D-I26:ICD-10-D-I2699
Pulmonary Embolism	ICD-9-D-4151:ICD-9-D-4159, ICD-10-D-I26:ICD-10-D-I269
Acute Myocardial Infarctions	ICD-9-D-410:ICD-9-D-41099, ICD-9-D-412:ICD-9-D-41299, ICD-10-D-I21:ICD-10-D-I2199, ICD-10-D-I22:ICD-10-D-I2299, ICD-10-D-I252
Acute Kidney Injury	ICD-9-D-5845, ICD-9-D-5846, ICD-9-D-5847, ICD-9-D-5848, ICD-9-D-5849, ICD-10-D-N17:ICD-10-D-N179
Transfusion	ICD-9-P-9904, ICD-10-P-3023, ICD-10-P-30230AZ, ICD-10-P-30230G0, ICD-10-P-30230G2, ICD-10-P-30230G3, ICD-10-P-30230G4, ICD-10-P-30230H0, ICD-10-P-30230H1, ICD-10-P-30230J0, ICD-10-P-30230J1, ICD-10-P-30230K0, ICD-10-P-30230K1, ICD-10-P-30230L0, ICD-10-P-30230L1, ICD-10-P-30230M0, ICD-10-P-30230M1, ICD-10-P-30230N0, ICD-10-P-30230N1, ICD-10-P-30230P0, ICD-10-P-30230P1, ICD-10-P-30230Q0, ICD-10-P-30230Q1, ICD-10-P-30230R0, ICD-10-P-

	30230R1, ICD-10-P-30230S0, ICD-10-P-30230S1, ICD-10-P-30230T0, ICD-10-P-30230T1, ICD-10-P-30230V0, ICD-10-P-30230V1, ICD-10-P-30230W0, ICD-10-P-30230W1, ICD-10-P-30230X0, ICD-10-P-30230X2, ICD-10-P-30230X3, ICD-10-P-30230X4, ICD-10-P-30230Y0, ICD-10-P-30230Y2, ICD-10-P-30230Y3, ICD-10-P-30230Y4, ICD-10-P-30233AZ, ICD-10-P-30233G0, ICD-10-P-30233G2, ICD-10-P-30233G3, ICD-10-P-30233G4, ICD-10-P-30233H0, ICD-10-P-30233H1, ICD-10-P-30233J0, ICD-10-P-30233J1, ICD-10-P-30233K0, ICD-10-P-30233K1, ICD-10-P-30233L0, ICD-10-P-30233L1, ICD-10-P-30233M0, ICD-10-P-30233M1, ICD-10-P-30233N0, ICD-10-P-30233N1, ICD-10-P-30233P0, ICD-10-P-30233P1, ICD-10-P-30233Q0, ICD-10-P-30233Q1, ICD-10-P-30233R0, ICD-10-P-30233R1, ICD-10-P-30233S0, ICD-10-P-30233S1, ICD-10-P-30233T0, ICD-10-P-30233T1, ICD-10-P-30233V0, ICD-10-P-30233V1, ICD-10-P-30233W0, ICD-10-P-30233W1, ICD-10-P-30233X0, ICD-10-P-30233X2, ICD-10-P-30233X3, ICD-10-P-30233X4, ICD-10-P-30233Y0, ICD-10-P-30233Y2, ICD-10-P-30233Y3, ICD-10-P-30233Y4, ICD-10-P-30240AZ, ICD-10-P-30240G0, ICD-10-P-30240G2, ICD-10-P-30240G3, ICD-10-P-30240G4, ICD-10-P-30240H0, ICD-10-P-30240H1, ICD-10-P-30240J0, ICD-10-P-30240J1, ICD-10-P-30240K0, ICD-10-P-30240K1, ICD-10-P-30240L0, ICD-10-P-30240L1, ICD-10-P-30240M0, ICD-10-P-30240M1, ICD-10-P-30240N0, ICD-10-P-30240N1, ICD-10-P-30240P0, ICD-10-P-30240P1, ICD-10-P-30240Q0, ICD-10-P-30240Q1, ICD-10-P-30240R0, ICD-10-P-30240R1, ICD-10-P-30240S0, ICD-10-P-30240S1, ICD-10-P-30240T0, ICD-10-P-30240T1, ICD-10-P-30240V0, ICD-10-P-30240V1, ICD-10-P-30240W0, ICD-10-P-30240W1, ICD-10-P-30240X0, ICD-10-P-30240X2, ICD-10-P-30240X3, ICD-10-P-30240X4, ICD-10-P-30240Y0, ICD-10-P-30240Y2, ICD-10-P-30240Y3, ICD-10-P-30240Y4, ICD-10-P-30243AZ, ICD-10-P-30243G0, ICD-10-P-30243G2, ICD-10-P-30243G3, ICD-10-P-30243G4, ICD-10-P-30243H0, ICD-10-P-30243H1, ICD-10-P-30243J0, ICD-10-P-30243J1, ICD-10-P-30243K0, ICD-10-P-30243K1, ICD-10-P-30243L0, ICD-10-P-30243L1, ICD-10-P-30243M0, ICD-10-P-30243M1, ICD-10-P-30243N0, ICD-10-P-30243N1, ICD-10-P-30243P0, ICD-10-P-30243P1, ICD-10-P-30243Q0, ICD-10-P-30243Q1, ICD-10-P-30243R0, ICD-10-P-30243R1, ICD-10-P-30243S0, ICD-10-P-30243S1, ICD-10-P-30243T0, ICD-10-P-30243T1, ICD-10-P-30243V0, ICD-10-P-30243V1, ICD-10-P-30243W0, ICD-10-P-30243W1, ICD-10-P-30243X0, ICD-10-P-30243X2, ICD-10-P-30243X3, ICD-10-P-30243X4, ICD-10-P-30243Y0, ICD-10-P-30243Y2, ICD-10-P-30243Y3, ICD-10-P-30243Y4, ICD-10-P-30250G0, ICD-10-P-30250G1, ICD-10-P-30250H0, ICD-10-P-30250H1, ICD-10-P-30250J0, ICD-10-P-30250J1, ICD-10-P-30250K0, ICD-10-P-30250K1, ICD-10-P-30250L0, ICD-10-P-30250L1, ICD-10-P-30250M0, ICD-10-P-30250M1, ICD-10-P-30250N0, ICD-10-P-30250N1, ICD-10-P-30250P0, ICD-10-P-30250P1, ICD-10-P-30250Q0, ICD-10-P-30250Q1, ICD-10-P-30250R0, ICD-10-P-30250R1, ICD-10-P-30250S0, ICD-10-P-30250S1, ICD-10-P-30250T0, ICD-10-P-30250T1, ICD-10-P-30250V0, ICD-10-P-30250V1, ICD-10-P-30250W0, ICD-10-P-30250W1, ICD-10-P-30250X0, ICD-10-P-30250X1, ICD-10-P-30250Y0, ICD-10-P-30250Y1, ICD-10-P-30253G0, ICD-10-P-30253G1, ICD-10-P-

	30253H0, ICD-10-P-30253H1, ICD-10-P-30253J0, ICD-10-P-30253J1, ICD-10-P-30253K0, ICD-10-P-30253K1, ICD-10-P-30253L0, ICD-10-P-30253L1, ICD-10-P-30253M0, ICD-10-P-30253M1, ICD-10-P-30253N0, ICD-10-P-30253N1, ICD-10-P-30253P0, ICD-10-P-30253P1, ICD-10-P-30253Q0, ICD-10-P-30253Q1, ICD-10-P-30253R0, ICD-10-P-30253R1, ICD-10-P-30253S0, ICD-10-P-30253S1, ICD-10-P-30253T0, ICD-10-P-30253T1, ICD-10-P-30253V0, ICD-10-P-30253V1, ICD-10-P-30253W0, ICD-10-P-30253W1, ICD-10-P-30253X0, ICD-10-P-30253X1, ICD-10-P-30253Y0, ICD-10-P-30253Y1, ICD-10-P-30260G0, ICD-10-P-30260G1, ICD-10-P-30260H0, ICD-10-P-30260H1, ICD-10-P-30260J0, ICD-10-P-30260J1, ICD-10-P-30260K0, ICD-10-P-30260K1, ICD-10-P-30260L0, ICD-10-P-30260L1, ICD-10-P-30260M0, ICD-10-P-30260M1, ICD-10-P-30260N0, ICD-10-P-30260N1, ICD-10-P-30260P0, ICD-10-P-30260P1, ICD-10-P-30260Q0, ICD-10-P-30260Q1, ICD-10-P-30260R0, ICD-10-P-30260R1, ICD-10-P-30260S0, ICD-10-P-30260S1, ICD-10-P-30260T0, ICD-10-P-30260T1, ICD-10-P-30260V0, ICD-10-P-30260V1, ICD-10-P-30260W0, ICD-10-P-30260W1, ICD-10-P-30260X0, ICD-10-P-30260X1, ICD-10-P-30260Y0, ICD-10-P-30260Y1, ICD-10-P-30263G0, ICD-10-P-30263G1, ICD-10-P-30263H0, ICD-10-P-30263H1, ICD-10-P-30263J0, ICD-10-P-30263J1, ICD-10-P-30263K0, ICD-10-P-30263K1, ICD-10-P-30263L0, ICD-10-P-30263L1, ICD-10-P-30263M0, ICD-10-P-30263M1, ICD-10-P-30263N0, ICD-10-P-30263N1, ICD-10-P-30263P0, ICD-10-P-30263P1, ICD-10-P-30263Q0, ICD-10-P-30263Q1, ICD-10-P-30263R0, ICD-10-P-30263R1, ICD-10-P-30263S0, ICD-10-P-30263S1, ICD-10-P-30263T0, ICD-10-P-30263T1, ICD-10-P-30263V0, ICD-10-P-30263V1, ICD-10-P-30263W0, ICD-10-P-30263W1, ICD-10-P-30263X0, ICD-10-P-30263X1, ICD-10-P-30263Y0, ICD-10-P-30263Y1, ICD-10-P-30273H1, ICD-10-P-30273J1, ICD-10-P-30273K1, ICD-10-P-30273L1, ICD-10-P-30273M1, ICD-10-P-30273N1, ICD-10-P-30273P1, ICD-10-P-30273Q1, ICD-10-P-30273R1, ICD-10-P-30273S1, ICD-10-P-30273T1, ICD-10-P-30273V1, ICD-10-P-30273W1, ICD-10-P-30277H1, ICD-10-P-30277J1, ICD-10-P-30277K1, ICD-10-P-30277L1, ICD-10-P-30277M1, ICD-10-P-30277N1, ICD-10-P-30277P1, ICD-10-P-30277Q1, ICD-10-P-30277R1, ICD-10-P-30277S1, ICD-10-P-30277T1, ICD-10-P-30277V1, ICD-10-P-30277W1, ICD-10-P-30280B1, ICD-10-P-30283B1

Table S3. Codes used to define THA joint complication outcomes

Criteria	Code(s)
Joint Complications	
All-cause Revision	CPT-27132, CPT-27134, CPT-27137, CPT-27138, CPT-11981, CPT-27091, CPT-20680, ICD-9-P-0070, ICD-9-P-0071, ICD-9-P-0072, ICD-9-P-0073, ICD-9-P-8153, ICD-9-P-8456, ICD-9-P-8457, ICD-10-P-0SW908Z, ICD-10-P-0SW909Z, ICD-10-P-0SW90BZ, ICD-10-P-0SW90JZ, ICD-10-P-0SW938Z, ICD-10-P-0SW93JZ, ICD-10-P-0SW94JZ, ICD-10-P-0SW9X8Z, ICD-10-P-0SW9XJZ, ICD-10-P-0SWA0JZ, ICD-10-P-0SWA4JZ, ICD-10-P-0SWAXJZ, ICD-10-P-0SWB08Z, ICD-10-P-0SWB09Z, ICD-10-P-0SWB0BZ, ICD-10-P-0SWB0JZ, ICD-

	10-P-0SWB3JZ, ICD-10-P-0SWB48Z, ICD-10-P-0SWB4JZ, ICD-10-P-0SWBX8Z, ICD-10-P-0SWBXJZ, ICD-10-P-0SWE0JZ, ICD-10-P-0SWE4JZ, ICD-10-P-0SWEXJZ, ICD-10-P-0SWR0JZ, ICD-10-P-0SWRXJZ, ICD-10-P-0SWS0JZ, ICD-10-P-0SWS3JZ, ICD-10-P-0SWS4JZ, ICD-10-P-0SWSXJZ
Periprosthetic Joint Fracture	ICD-9-D-99644, ICD-10-D-M9701XA, ICD-10-D-M9702XA, ICD-10-D-T84040A, ICD-10-D-T84041A
Prosthetic Joint Infection	ICD-10-D-T8450XA, ICD-10-D-T8459XA, ICD-9-D-99666
Aseptic Loosening	ICD-9-D-99641, ICD-10-D-T84030A, ICD-10-D-T84031A
Prosthetic Dislocation	ICD-9-D-99642, ICD-10--T84020A, ICD-10-D-T84021A

Table S4. Codes used to define TKA joint complication outcomes

Criteria	Code(s)
Joint Complications	
Manipulation under anesthesia/Stiffness	CPT-27570, CPT-29884
Septic Revision	CPT-27487, ICD-9-P-0080, ICD-10-P-0SWC0JZ, ICD-10-P-0SWCXJZ, ICD-10-P-0SWD0JZ, ICD-10-P-0SWDXJZ<br>+ ICD-9-D-99666, ICD-10-D-M01X61, ICD-10-D-M01X62, ICD-10-D-M01X69, ICD-10-D-T8453XA, ICD-10-D-T8453XD, ICD-10-D-T8453XS, ICD-10-D-T8454XA, ICD-10-D-T8454XD, ICD-10-D-T8454XS<br>+ CPT-27488, ICD-9-P-8006, ICD-10-P-0SPC0JZ, ICD-10-P-0SPD0JZ<br>+ CPT-11981, ICD-9-P-8456, ICD-10-P-0SHC08Z, ICD-10-P-0SHD08Z, ICD-10-P-0SRC0EZ, ICD-10-P-0SRD0EZ<br>+ CPT-27447, ICD-9-P-8154, ICD-10-P-0SRC0J9, ICD-10-P-0SRC0JA, ICD-10-P-0SRC0JZ, ICD-10-P-0SRD0J9, ICD-10-P-0SRD0JA, ICD-10-P-0SRD0JZ<br>+ CPT-11982, ICD-9-P-8457, ICD-10-P-0SPC08Z, ICD-10-P-0SPC0EZ, ICD-10-P-0SPD08Z, ICD-10-P-0SPD0EZ
Aseptic Revision	CPT-27486, CPT-27487, ICD-9-P-0080, ICD-9-P-0081, ICD-9-P-0082, ICD-9-P-0083, ICD-9-P-0084, ICD-10-P-0SWC0JZ, ICD-10-P-0SWD0JZ, ICD-10-P-0SWC0JC, ICD-10-P-0SWCXJZ, ICD-10-P-0SWD0JC, ICD-10-P-0SWV0JZ, ICD-10-P-0SWDXJZ, ICD-10-P-0SWW0JZ, ICD-10-P-0SWC09Z, ICD-10-P-0SWT0JZ, ICD-10-P-0SWD09Z, ICD-10-P-0SWU0JZ
Knee Periprosthetic Fracture	ICD-9-D-99644, ICD-10-D-M9711XA, ICD-10-D-M9712XA, ICD-10-D-T84042A, ICD-10-D-T84043A
Loosening	ICD-9-D-99641, ICD-10-D-T84032A, ICD-10-D-T84033A
